# Decitabine demonstrates antileukemic activity in B cell precursor acute lymphoblastic leukemia with MLL rearrangements

**DOI:** 10.1186/s13045-018-0607-3

**Published:** 2018-05-04

**Authors:** C. Roolf, A. Richter, C. Konkolefski, G. Knuebel, A. Sekora, S. Krohn, J. Stenzel, B. J. Krause, B. Vollmar, H. Murua Escobar, C. Junghanss

**Affiliations:** 1 Department of Medicine, Clinic III – Hematology, Oncology, Palliative Medicine, Rostock University Medical Center, Ernst-Heydemann-Str. 6, 18057 Rostock, Germany; 2 Department of Nuclear Medicine, Rostock University Medical Center, University of Rostock, Gertrudenplatz 1, 18057 Rostock, Germany; 3 Institute of Experimental Surgery, Rostock University Medical Center, University of Rostock, Schillingallee 69a, 18057 Rostock, Germany

**Keywords:** Acute lymphoblastic leukemia, In vivo imaging, Bioluminescence, PET/CT, Patient-derived xenograft model

## Abstract

**Background:**

Promotor hypermethylation of CpG islands is common in B cell precursor acute lymphoblastic leukemia (BCP-ALL) with mixed lineage leukemia (MLL) gene rearrangements. Hypomethylating agents (HMA) such as azacitidine (AZA) and decitabine (DEC) reduce DNA hypermethylation by incorporation into DNA and were successfully introduced into the clinic for the treatment of myeloid neoplasias.

**Methods:**

Here, we investigated whether HMA induce comparable biological effects in MLL-positive BCP-ALL. Further, efficacy of HMA and concomitant application of cytostatic drugs (cytarabine and doxorubicin) were evaluated on established SEM and RS4;11 cell lines. In addition, promising approaches were studied on BCP-ALL cell line- and patient-derived xenograft models.

**Results:**

In general, DEC effects were stronger compared to AZA on MLL-positive BCP-ALL cells. DEC significantly reduced proliferation by induction of cell cycle arrest in G0/G1 phase and apoptosis. Most sensitive to HMA were SEM cells which are characterized by a fast cell doubling time. The combination of low-dose HMA and conventional cytostatic agents revealed a heterogeneous response pattern. The strongest antiproliferative effects were observed when ALL cells were simultaneously exposed to HMA and cytostatic drugs. Most potent synergistic effects of HMA were induced with cytarabine. Finally, the therapeutic potential of DEC was evaluated on BCP-ALL xenograft models. DEC significantly delayed leukemic proliferation in xenograft models as demonstrated longitudinally by non-invasive bioluminescence as well as ^18^F-FDG-PET/CT imaging. Unexpectedly, in vivo concomitant application of DEC and cytarabine did not enhance the antiproliferative effect compared to DEC monotherapy.

**Conclusions:**

Our data reveal that DEC is active in MLL-positive BCP-ALL and warrant clinical evaluation.

**Electronic supplementary material:**

The online version of this article (10.1186/s13045-018-0607-3) contains supplementary material, which is available to authorized users.

## Background

B cell precursor acute lymphoblastic leukemia (BCP-ALL) is characterized by several molecular and cytogenetic changes. One of the most frequently involved genetic alterations in BCP-ALL is the rearrangement of the mixed lineage leukemia (MLL) gene. Thereby, the t(4;11)(q21;q23)/MLL-AF4 chromosomal translocation is the second most frequent translocation in adult ALL overall [1]. MLL-AF4-positive ALL is generally considered as high-risk leukemia associated with poor clinical outcome [2]. Therefore, in general, multidrug chemotherapy regimens are used for remission induction and consolidation [3–5]. In the case of CD20 positivity, antibody-based anti-CD-20 immunotherapy has proved to be beneficial [6]. Subsequent allogeneic stem cell transplantation is recommended. No subtype-specific targeted therapy for MLL patients has been established yet [2]. The exact pathogenesis of MLL-positive ALL has not yet been fully understood. However, epigenetic dysregulation and the acquisition of additional secondary genetic mutations seem to play a pivotal role in MLL-driven leukemogenesis [7].

Epigenetic dysregulation appears to be prevalent in MLL-positive leukemia, and specific methylation patterns have been reported [8, 9]. Infant MLL-rearranged ALL is characterized by aberrant promotor hypermethylation in CpG islands of tumor suppressor genes inducing transcriptional silencing [10]. Thereby, key signaling pathways influencing cell cycle progression, apoptosis, DNA repair, or cell differentiation are dysregulated and have therefore been proposed to be major factors in the development of MLL-ALL.

In general, hypermethylated genes can be targeted by hypomethylating agents (HMA) such as cytosine analogs azacitidine (AZA) or decitabine (DEC). These agents inhibit the function of DNA methyltransferases (DNMT) by incorporation into the DNA and prevent the methylation of cytosine during cell division, resulting in genome-wide demethylation [11]. Both drugs are used in the treatment of acute myeloid leukemia (AML) [12, 13].

The efficacy of HMA on BCP-ALL has not yet been investigated in detail. So far, DEC has been studied in two small clinical trials in relapsed and refractory B-ALL patients [14, 15]. Both studies demonstrated clinical activity and DNA demethylation. The overall response rate was higher when DEC was given in combination with a commonly used chemotherapy regimen [14]. Additionally, combination with the histone deacetylase inhibitor Vorinostat followed by standard re-induction chemotherapy demonstrated clinical benefit in relapsed ALL patients [15]. To date, clinical trials with AZA have not been implemented in ALL. In vitro, AZA in combination with histone deacetylase inhibitor Panobinostat was reported to induce synergistic antiproliferative effects in ALL cell lines [16].

Here, we hypothesized that HMA exhibit antiproliferative effects depending on drug exposition sequence in MLL-positive BPC-ALL. Further, we hypothesized that HMA increases the sensitivity to concomitant cytostatic agents. In order to proof our hypotheses, cell culture and xenograft models of BCP-ALL were used. Thereby, in vivo ALL cell expansion was studied with non-invasive imaging technologies using bioluminescence and PET/CT.

## Methods

### Cell lines and cell culture

The human BCP-ALL cell lines SEM and RS4;11 carry the translocation t(4;11) and were purchased from DSMZ (Braunschweig, Germany). Cells were cultured as previously described [17]. Briefly, cells were maintained as suspension cultures in Iscove’s MDM (SEM) or alpha-MEM (RS4;11) supplemented with 10% heat-inactivated fetal bovine serum (Biochrom, Berlin, Germany) and 100 μg/ml penicillin and streptomycin (Biochrom) at 37 °C in humidified air containing 5% CO_2_. Cell doubling time for SEM has been described earlier to be 30 h and for RS4;11 60 h [18, 19]. Our analysis revealed slightly longer doubling times (i.e., SEM: 33-36 h and RS4;11: 51-56 h).

### Patients

Mononuclear cells of bone marrow (BM) aspirates were obtained from three newly diagnosed ALL patients (Rostock University Medical Center, Germany) and isolated by density centrifugation. Cancer hotspot mutations were analyzed with next-generation sequencing (Ion PGM System, Thermo Fisher Scientific, Schwerte, Germany) according to the manufacturer’s protocol. Patient’s characteristics are summarized in Additional file 1. The study was performed in accordance to the Declaration of Helsinki and the local ethical standards of the Rostock University Medical Center.

### Drugs

AZA and DEC were purchased from Selleckchem (Munich, Germany). Cytarabine (AraC) and doxorubicin (Doxo) were purchased from CellPharm GmbH (Bad Vilbel, Germany). Control cells were cultured in their medium containing DMSO in the same concentration as present in the drug-treated cells. For xenograft studies, DEC was dissolved in PBS.

### Inhibition experiments and drug combination studies

Cells with a density of 0.33 × 10^6^/ml were incubated with serial HMA dilutions for up to 72 h. Subsequently, low-dose HMA were combined with low dose of AraC or Doxo. Cytostatics were added at the time of cell seeding either simultaneously, 24 h before, or 24 h after HMA application. The used drug concentrations can be achieved in human plasma [20, 21]. All experiments were carried out in biological triplicates.

### Study of proliferation and metabolic activity

Proliferation was assessed by counting viable cells using trypan blue dye exclusion. Metabolic activity was evaluated using WST-1 assay (Roche, Mannheim, Germany) [22].

Analyses of cell cycle and apoptosis were performed as previously described [22].

### Methylation specific quantitative PCR (MSqPCR)

*CDH13* and *LINE-1* methylation was quantified by MSqPCR (Additional files 2 and 3).

### Generation of GFP- and ffluc-expressing cells

SEM and RS4;11 were stably transduced with enhanced firefly luciferase (ffluc) which was subcloned into the multicloning site of the pCDH-EF1-MCS-T2A-copGFP vector (System Biosciences, Mountain View, CA, USA) using EcoRI and BamHI [23].

### Xenograft mouse model

NOD scid gamma mice (NSG, Charles River Laboratories, Sulzfeld, Germany) were bred and housed under specific pathogen-free conditions. NSG mice (10–16 weeks old) were intravenously injected with 2.5 × 10^6^ SEM-ffluc-GFP, RS4;11-ffluc-GFP, or de novo BCP-ALL cells.

Tumor burden was assessed by bioluminescence imaging (BLI) using NightOWL LB983 in vivo imaging system and Indigo software version 1.04 (Berthold Technologies, Bad Wildbach, Germany). Animals were intraperitoneally injected with 4.5 mg d-luciferin (Goldbiotechnology, St. Louis, USA). Mice were imaged 10 min after luciferin injection in prone and supine position for 60-s exposure time (sample size 150 × 20 mm; binning 4 × 4; emission 560 nm). BLI signals (ph/s) were calculated as the sum of both prone and supine acquisitions for each mouse.

Treatment started 7 days after tumor cell injection when BLI revealed equal engraftment of leukemia cells in all mice. Mice were treated intraperitoneally with a vehicle (isotonic saline: d7–d10), daily with 0.4 mg/kg BW DEC (d7–d10), daily with 150 mg/kg BW AraC (d7, d8), or both [24, 25]. Each group comprised of nine mice (Additional files 4 and 5).

Drug response was evaluated weekly using flow cytometry analyses (peripheral blood (PB)) and whole body BLI (ffluc) for up to 30 days. Mice were sacrificed, and cell suspensions were prepared from spleen and BM as previously reported [26].

Patient-derived xenograft (PDX) mice were treated as described above. Treatment response was analyzed by measuring frequency of human CD19 (clone 4G7, BD, Heidelberg, Germany) and human CD45 (clone 2D1, BD) in blood (weekly) and BM and spleen (both after euthanasia).

All experiments were approved by the review board of the federal state of Mecklenburg-Vorpommern, Germany (reference number: LALLF MV/7221.3-1.1-002/15).

### ^18^F-FDG-PET/CT imaging

^18^F-FDG was injected into the tail vein with 18.4 ± 2.1 MBq (distribution time 60 min). Imaging was performed for 15 min static acquisition and later analyzed (Inveon PET/CT Siemens, Knoxville, TN, USA). ^18^F-FDG uptake in spleen was determined by percent intensity of the injected dose per g (%ID/g). To calculate the metabolic volume of the spleen, 70% of measured %ID/g_max_ of the spleen was set as threshold.

### Statistical analysis

Results within each experiment were described using mean and standard deviation. Significance between strains was calculated using Student’s *t* test (Microsoft excel software, version 2010, München, Germany). A *p* value < 0.05 was considered to be significant. Bliss independence model is widely used to determine effects of the drug combinations. Drug combination effects were obtained by the difference (Δ) between the observed (*O*) and the expected (*E*) inhibition of combined treatment. *E* is calculated as follows: *E* = (*A* + *B*) − (A*B), where *A* and *B* are the relative inhibition of single agent *A* and *B*. Δ > 0 indicating synergistic, and Δ <  0 antagonistic effects [27]. For the calculation, mean values of the metabolic activity or mean values of proliferation from three independent experiments were used.

## Results

### HMA inhibit proliferation and metabolic activity

**Fig. 1:**
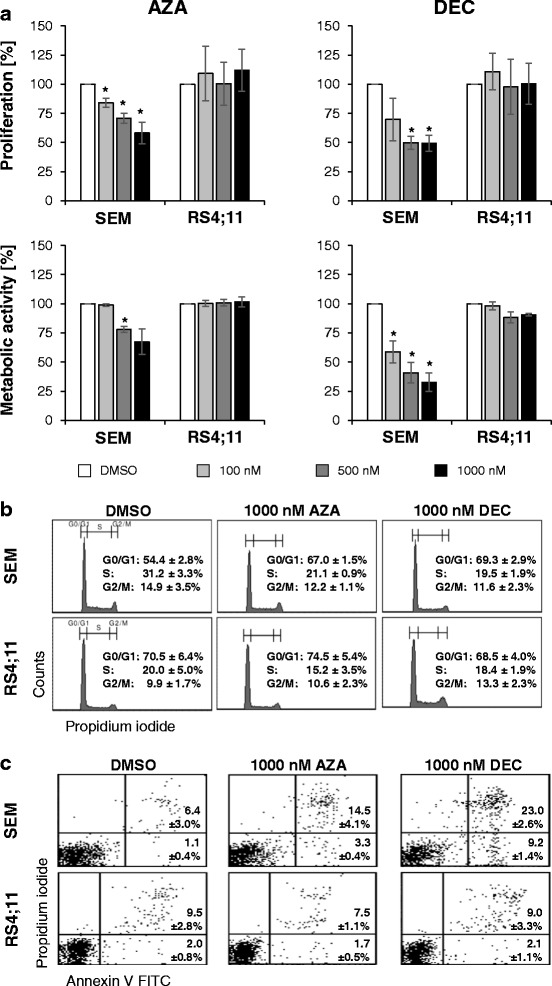
HMA interfere with biological cell functions. SEM and RS4;11 cells were exposed to HMA for up to 72 h. Results were expressed as a percentage of DMSO-treated control cells and displayed as mean ± SD of three independent experiments. Significant treatment effects vs. DMSO are labeled with * (*p* < 0.05). **a** Proliferation and metabolic activity of SEM cells were significantly reduced after AZA and DEC exposure. HMA did not influence proliferation or metabolic activity of RS4;11 cells. **b** HMA induced an increase of SEM cells in G_1_/G_0_ phase, with a decreased number of cells in S phase. **c** In SEM cells, HMA increase the amount of early apoptotic and late apoptotic cells compared to DMSO-treated cells. Effect of DEC treatment was stronger than the effect of AZA treatment. No induction of apoptosis was observed in RS4;11 cells

Effects of AZA and DEC were analyzed in SEM and RS4;11 cells at various concentrations (100–1000 nM) (Fig. 1a). A dose-dependent effect of HMA on proliferation and metabolic activity was observed in SEM cells after 72-h drug exposure. Cell proliferation was reduced to 58.1% (1000 nM AZA) and to 49.3% (1000 nM DEC) compared to control cells (=100%). Metabolic activity decreased significantly by AZA up to 67.5% and by DEC up to 32.7% compared to control (100%). In RS4;11, HMA induced no significant effects on proliferation or metabolic activity. Cell numbers are displayed in Additional file 6.

Furthermore, cell cycle analysis revealed a G_0_/G_1_ arrest in HMA-treated SEM cells (Fig. 1b). Here, HMA increased the number of cells in G_0_/G_1_ phase significantly after 72 h (1000 nM AZA: 67.0%, 1000 nM DEC: 69.3% vs. control: 54.4%) and decreased the amount of cells in M phase. In RS4;11, distribution of cell cycle phases was not influenced by HMA.

Treatment with HMA induced apoptosis in SEM cells (Fig. 1c). The amount of apoptotic cells increased up to 17.8% (1000 nM AZA) or up to 32.2% (1000 nM DEC). Apoptosis rates remained unchanged in RS4;11 cells after HMA exposure.

In summary, SEM cells were more sensitive to HMA than RS4;11 cells. Effects of DEC were stronger than those of AZA.

### DEC diminishes methylation levels

Cadherin 13 (CDH13), a member of the cadherin superfamily, is frequently hypermethylated in various types of cancer including BCP-ALL and was selected to evaluate CpG demethylating effects of HMA [28]. Changes on global DNA methylation were examined with the long interspersed element 1 (LINE-1) [29]. Methylation status was analyzed for up to 48 h by MSqPCR (Additional file 3). Methylation of LINE-1 or CDH13 was not modulated after short-time HMA exposure (0.5–24 h) (data not shown). Incubation with 1000 nM DEC for 48 h resulted in significantly decreased methylation of LINE-1 (67.7 ± 1.8%) as well as significant modulation of CDH13 (93.1 ± 0.7%) in SEM cells compared to DMSO-treated controls (considered as 100%). No significant changes on DNA methylation of CDH13 or LINE-1 occurred in SEM cells after AZA exposure. In RS4;11, methylation of LINE-1 and CDH13 was not affected by both substances.

### Drug combination studies—influence of exposure sequence

Active drugs in ALL include cell cycle influencing agents and topoisomerase inhibitors. As HMA induce broad effects on a variety of genes (e.g., cell cycle), the sequence of drug exposure might be of importance. Therefore, sequential applications of low-dose HMA and conventional cytostatics were analyzed.

#### HMA and AraC

**Fig. 2:**
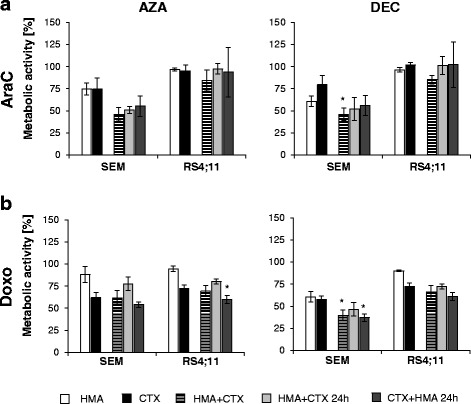
Effects of HMA and cytostatic drug combinations. Cells were treated with HMA in the absence or presence of AraC (**a**) or Doxo (**b**). Cytostatic drugs (CTX) were added simultaneously, 24 h before, or 24 h after treatment with HMA. CTX concentrations were based on low doses and used as follows: 100 nM AZA (SEM), 500 nM AZA (RS4;11), 100 nM DEC (SEM), 500 nM DEC (RS4;11); 10 nM AraC (SEM); 2500 nM AraC (RS4;11), 12.5 nM Doxo (SEM, RS4;11). The metabolic activity was determined 72 h after drug application. Significant effects are marked with * when metabolic activity decreased significantly (*p* < 0.05) compared at once to DMSO, HMA, and CTX

The combination of HMA and AraC enhanced the antiproliferative effect in SEM cells (Fig. 2, Additional file 7). Simultaneous DEC and AraC application decreased metabolic activity significantly (46.0 ± 7.1%) compared to control (100%) and single treatment with DEC (60.7 ± 5.9%) or AraC (79.5 ± 10.5%). Also, the simultaneous application of AraC with AZA diminished metabolic activity (46.1 ± 7.7%). However, the differences were not statistically significant compared to AraC alone. Sequential drug applications did not increase the sensitivity of AraC-exposed SEM cells compared to simultaneous treatment.

RS4;11 cells were not sensitive to AraC and were incubated with a 250-fold higher concentration compared to SEM. Additional exposure of HMA (simultaneous or sequential) did not increase the sensitivity to AraC treatment.

In addition, analysis of drug interaction with Bliss independence model was performed and showed that drugs act synergistically when cells were simultaneously exposed to HMA and AraC (Table 1). Synergistic effects were calculated for proliferation and metabolic activity. Antagonistic effects were induced by sequential drug exposure.

**Table 1:** Analyses of HMA combinations with AraC on BCP-ALL cells

		Drug combination effect					
		AZA + AraC	AZA + AraC (24 h)	AraC + AZA (24 h)	DEC + AraC	DEC + AraC (24 h)	AraC + DEC (24 h)
SEM	Metabolism	0.095	0.045	0.004	0.022	− 0.039	− 0.077
	Proliferation	0.064	0.006	0.001	0.017	− 0.048	0.041
RS4;11	Metabolism	0.076	− 0.055	− 0.018	0.128	− 0.032	− 0.043
	Proliferation	0.016	0.044	− 0.003	0.000	0.025	0.076

Drug combination effect: difference between the observed and the expected inhibition (*E*) of combined treatment. *E* is calculated as follows: *E* = (*A* + *B*) − (A*B); *A* is inhibition effect of drug A; *B* inhibition effect of drug B values > 0: synergistic, values < 0: antagonistic

#### HMA and Doxo

Exposure of BCP-ALL cells to Doxo and HMA induced in part significant antiproliferative effects compared to the monoapplication (Fig. 2, Additional file 7). Metabolism in SEM cells decreased significantly to 39.5 ± 6.0% when DEC and Doxo were added simultaneously and to 37.0 ± 4.4% when Doxo was given 24 h in advance of DEC (DEC 60.7 ± 5.9%; Doxo 57.5 ± 3.9%). But these effects were not synergistic as demonstrated by Bliss statistic (Table 2).

**Table 2:** Analyses of HMA combinations with Doxo on BCP-ALL cells

		Drug combination effect					
		AZA + Doxo	AZA + Doxo (24 h)	Doxo + AZA (24 h)	DEC + Doxo	DEC + Doxo (24 h)	Doxo + DEC (24 h)
SEM	Metabolism	− 0.071	− 0.230	0.006	− 0.046	− 0.117	− 0.021
	Proliferation	− 0.002	− 0.192	0.015	− 0.014	− 0.094	− 0.042
RS4;11	Metabolism	− 0.009	− 0.118	0.086	− 0.007	− 0.071	0.044
	Proliferation	− 0.022	− 0.227	0.016	− 0.028	− 0.301	0.011

Drug combination effect: difference between the observed and the expected inhibition (*E*) of combined treatment. *E* is calculated as follows: *E* = (*A* + *B*) − (A*B); *A* is inhibition effect of drug A; *B* inhibition effect of drug B values > 0: synergistic, values < 0: antagonistic

RS4;11 cells showed a significant decreased metabolism when Doxo was given 24 h in advance to AZA (59.9 ± 4.4%) while delayed Doxo application resulted in opposite effects (80.3 ± 2.8%). Synergism as well as antagonism has also been confirmed by Bliss.

In summary, pronounced effects were observed when cells were simultaneously exposed to HMA and cytotoxic agents. Pre-treatment with HMA was less effective and showed no beneficial effect in vitro.

### DEC demonstrates antileukemic activity in vivo

**Fig. 3:**
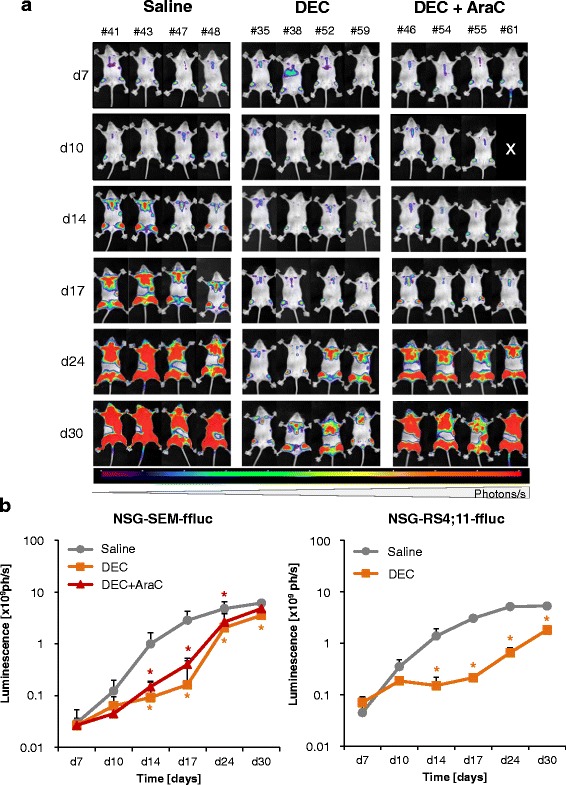
DEC decelerates ALL engraftment in vivo*.* **a** Demonstration of in vivo monitoring of luciferase expression after injection of luciferase and monitored by BLI (ph/s) in SEM-ffluc xenograft mice. Demonstrated are mice after treatment with saline-, DEC- and DEC + AraC-treated mice during 30 days (four representative mice per group). DEC-treated mice show decelerated leukemia cell proliferation as indicated with a lower BLI signal compared to saline-treated mice. **b** Quantification of BLI signals (ph/s) was performed by adding whole body luminescence signals of prone and supine acquisition. BLI signals are summarized as mean ± SD for SEM-ffluc (saline: *n* = 9, DEC: *n* = 9, DEC + AraC: *n* = 9)- and RS4;11-ffluc (saline: *n* = 9, DEC: *n* = 9)-derived xenografts. Significant treatment effects are labeled with * (*p* < 0.05)

**Fig. 4:**
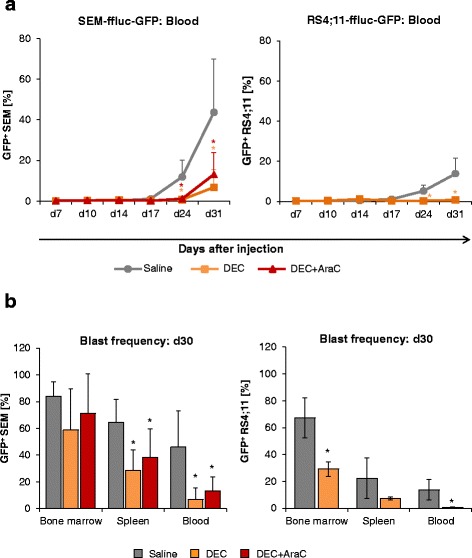
DEC decelerates blast frequency in BCP-ALL xenograft models. In vivo efficacy of DEC and DEC + AraC was investigated with flow cytometry of blood, BM, and spleen in SEM-ffluc and RS4;11-ffluc xenografts. **a** The longitudinal mean leukemic blast frequency (%GFP^+^) in blood after treatment is summarized for SEM-ffluc and RS4;11-ffluc. Each group comprises of nine mice. **b** At day 30, blast frequency (%GFP^+^) in BM, spleen, and blood is displayed for DEC-, DEC + AraC-, and saline-treated mice

Efficacy of DEC was investigated in an orthotopic ALL xenograft mouse model (SEM-ffluc-GFP, RS4;11-ffluc-GFP). Treatment started 7 days post-injection if a tumor activity was detectable by BLI. Therapy response was investigated longitudinally (Fig. 3). Additionally, the amount of GFP-expressing leukemic cells in PB (Fig. 4a, b) was monitored.

DEC significantly delayed leukemia cell proliferation in SEM-ffluc- and RS4-ffluc-derived xenograft models compared to saline-treated mice (Fig. 3). Proliferation differences between saline- and treated-mice were significant starting on day 14.

Further, SEM-ffluc mice were treated with DEC and AraC in combination. Of interest, additional treatment with AraC did not intensify the DEC-induced effect. Unexpectedly, DEC-treated mice exhibited a lower tumor load compared to DEC + AraC-treated mice albeit not significant.

This was also confirmed by analysis of blood, BM, and spleen (Fig. 4). On day 24, leukemic blast frequency in the blood (% SEM-ffluc-GFP) was detectable in saline-treated mice and significantly decreased in SEM-ffluc DEC-treated mice (DEC: 0.8 ± 0.4%; DEC + AraC: 1.2 ± 0.7% vs. saline-treated: 11.9 ± 8.3%). Comparable effects were observed in spleen and BM at day 30 (Fig. 4b). In total, nine animals died unexpected (Additional files 4 and 5). To summarize, DEC treatment did not eradicate ALL but delayed disease progression in both xenograft models.

### Decitabine reduces metabolic activity

**Fig. 5:**
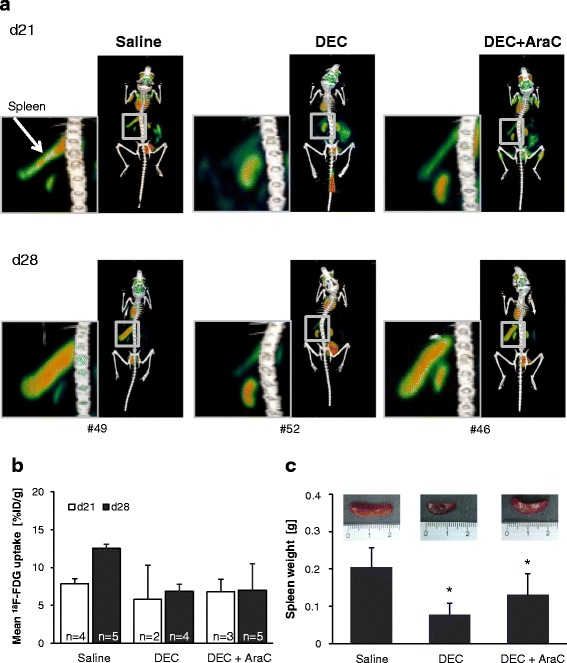
^18^F-FDG uptake in spleen is decreased after DEC treatment. **a** PET/CT was performed on days 21 and 28 in SEM-ffluc xenografts. Representative PET/CT scans demonstrate differences on ^18^F-FDG uptake. ^18^F-FDG uptake in spleens of DEC-treated mice was lower than in controls. **b** ^18^F-FDG uptake in spleen was calculated for all mice and is expressed as mean [%ID/g] of the metabolic volume. Results are summarized as mean ± SD. The number of analyzed mice for each treatment group is indicated in the bars. **c** Displayed are representative images of spleen from saline-, DEC- and DEC + AraC-treated group; spleen weight is summarized as mean ± SD

Metabolic activity can be evaluated with PET/CT analyzing glucose uptake after ^18^F-FDG tracer injection. Here, we applied this approach successfully to ALL cell imaging in xenograft mice. ^18^F-FDG uptake was monitored on d21 and d28 after inoculation of SEM-ffluc GFP cells into NSG mice (Fig. 5a). Physiologic ^18^F-FDG tracer uptake was detected in all animals (heart, bladder, kidney, brain). Metabolic active ALL cells were depicted by ^18^F-FDG accumulation in spleen and were quantifiable (Fig. 5b). At day 21, metabolic active ALL cells were detectable in spleen of controls (7.9 ± 0.7% ID/g) and DEC-treated (5.8 ± 4.5% ID/g) and AraC + DEC-treated (6.8 ± 1.7% ID/g) mice. At day 28, ^18^F-FDG uptake increased in controls (12.6 ± 0.5% ID/g) whereas only marginally changed in DEC (6.9 ± 0.9% ID/g)- or DEC + AraC (7.0 ± 3.5% ID/g)-treated mice.

In addition, metabolic tumor volume (mm^3^) derived by PET and metabolic maximum (% ID/g) were analyzed (Additional file 8). In saline-treated mice, the metabolic tumor volume increased over time from 52.3 ± 19 mm^3^ (d21) to 86.4 ± 21.1 mm^3^ (d28) whereas in DEC-treated mice, a decrease of metabolic tumor volume occurred from 42.7 ± 4.1 mm^3^ to 22.8 ± 15.5 mm^3^. Consistently, spleen weight differed significantly between saline- and DEC-treated groups (Fig. 5c).

### DEC reduces leukemic proliferation in de novo pro-B-ALL-derived xenografts

**Fig. 6:**
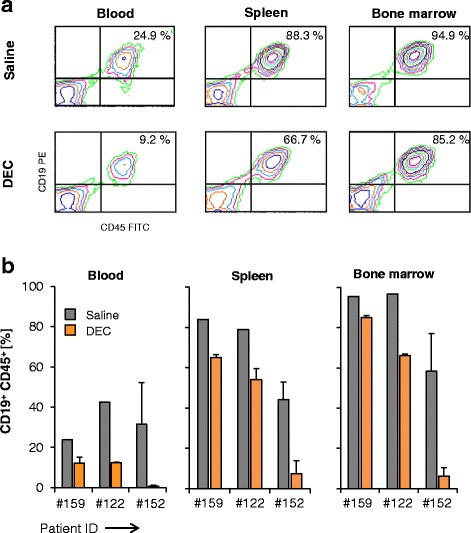
Blast frequency is lower in DEC-treated PDX models. **a** Analyses of human leukemia cells in BM, spleen, and blood analyzed from PDX mice (patient #159) by flow cytometry. Displayed are representative contour plots from saline (PDX-26)- and DEC (PDX-27)-treated mice. **b** Displayed is the blast frequency (%CD19^+^ and %CD45^+^) in BM, spleen, and blood of saline (#122: *n* = 1; #152: *n* = 2, #159: *n* = 1)- and DEC (#122: *n* = 2; #152: *n* = 2, #159: *n* = 2)-treated PDX mice

DEC therapy responses in PDX models generated from three individual adult BCP-ALL patients harboring MLL rearrangements was investigated. All patients had individual cancer mutations including TP53. Primary ALL cells were not stably transduced with GFP and ffluc vector. In line with our cell line-derived xenograft models, therapy started at day 7 after tumor cell injection. Maximal four mice were used for each individual patient sample. Therapy response was analyzed in PB for up to 53 days (range 29 to 53 days) depending on ALL proliferation in mice (Fig. 6a). Mice were sacrificed when leukemic blast frequency passed the 10% threshold in PB of saline-treated animals. The amount of leukemic cells in PB was markedly reduced in DEC-treated mice (range 0.5 to 15.2%) compared to controls (range 11.1 to 52.3%). Similarly, blast frequency in spleen and BM of DEC-treated PDX mice were lower than in controls (Fig. 6b).

Response to DEC was best in PDX mice derived from patient #152. Here, blast frequency in BM (6.3 ± 4.1%), spleen (7.5 ± 6.5%), and PB (0.8 ± 0.3%) in DEC-treated mice was lower compared to PDX from patients #159 (BM 84.7 ± 0.5%; spleen 66.6 ± 0.1%; PB 12.2 ± 3.0%) and #122 (BM 66.1 ± 0.8%; spleen 54.3 ± 5.3%; PB 12.4 ± 0.4%).

## Discussion

DNA hypermethylation is frequently observed in many neoplasms and is therefore a promising therapeutic target. Furthermore, it has been demonstrated that MLL-positive BCP-ALL displays a hypermethylated CpG promotor pattern providing a rationale for the evaluation of HMA approaches [10].

The aim of the current study was to evaluate the biological effect of HMA in MLL-positive BCP-ALL. Thereby, efficacy of drugs was analyzed in monoapplication and combination with conventional cytostatic drugs. Our results show that HMA decreased cell proliferation and viability of BCP-ALL. The combination of HMA with conventional cytostatic drugs revealed heterogeneous responses.

Two MLL-positive BCP-ALL cell lines (SEM and RS4;11) with different cell doubling times were selected as cell line-based models as they represent an ALL subtype-specific CpG island hypermethylation profile [10].

In SEM cells, a significant decrease of proliferation and metabolism after HMA exposure was demonstrated being associated with induction of cell cycle arrest and apoptosis. Interestingly, SEM cells were more sensitive to DEC compared to AZA exposure. In RS4;11 cells, no HMA induced significant biological effects. This could be explained on the one hand by the longer cell doubling time of RS4;11 compared to SEM cells because incorporation of HMA into the DNA occurred during DNA synthesis. On the other hand, the sensitivity of AZA and DEC can be explained by the fast decomposition of the compounds [30]. Stresemann et al. demonstrated that chemical stability of these compounds is dependent on pH value and temperature. At 37 °C, the half-life was 7 h for AZA and 21 h for DEC [30]. In addition, Leonard et al. investigated prolonged DEC exposure times on AML cells. The strongest antiproliferative effects were observed with repeated applications [31]. Thus, enhanced anti-proliferative effects in BCP-ALL cells in vitro might be stronger considering the half-life of AZA and DEC by using repeated HMA applications.

It is known that dosing regimen is critical for HMA. For AZA, several clinical trials have been reported with different time schedules and dosage [32–34]. We cannot exclude that repeated application or higher concentration of AZA affects methylation levels on LINE-1 and CDH13.

Longer incubation time and additional application of HMA might be required to sensitize RS4;11 cells and have to be evaluated in the future.

Effects of HMA on viability of B- and T-ALL cells were investigated in several previous studies [35–39]. Stumpel et al. analyzed the in vitro sensitivity of SEM and RS4;11 cells to different HMA. The authors showed that both cell lines were likewise DEC sensitive with IC50 values below 1 μM [38]. These observations are in line with our results. Shi et al. investigated HMA in T-ALL and reported increasing apoptosis rates, when DEC and a deacetylase inhibitor were combined [35].

Further, we investigated the sensitivity of BCP-ALL cells in regard to different HMA and chemotherapeutic drug exposition sequences. Drug doses for combination studies were chosen at low concentrations. We postulated that the order influences the extent of the observed effects. Our results indicate that the combination of low-dose HMA with low-dose conventional cytostatic drugs induced in part significantly stronger antiproliferative effects compared to single drug exposure. However, effects differed between cell lines and cytostatic drugs. Pronounced effects were observed in the simultaneous application approach. To our knowledge, we investigated for the first time biological effects on MLL-positive BCP-ALL cells in regard to sequence of drug exposure including HMA. In previous studies, it has been shown that HMA influenced chemosensitivity of ALL cells differently [36, 40]. Lu et al. investigated HMA effects on a panel of T-ALL cells [36]. They demonstrated synergistic as well as antagonistic effects when cells were exposed sequentially with DEC followed by application of prednisolone, etoposide, or AraC. Interestingly, in the same study, most pronounced effects were observed on RS4;11 cells. Strong synergistic effects were induced after pretreatment of DEC followed by AraC exposure [36]. Again, drug concentrations might explain differences to our results. Another preclinical study on BCP-ALL cells demonstrated that DEC pretreatment followed by prednisolone enhanced the cytotoxicity compared to DEC alone [40]. However, the authors did not investigate other sequences.

So far, DEC-induced effects on MLL-positive BCP-ALL xenograft models have not been investigated. Here, we validated the efficacy of DEC treatment by using non-invasive imaging techniques. Orthotopic ALL xenograft models have been successfully established to evaluate leukemic proliferation in vivo and offer a powerful tool for preclinical investigations [23, 41]. Engraftment of leukemic cells using BLI is easily detectable and allows a non-invasive longitudinal determination of leukemia burden [42].

Interestingly, a significant inhibition of leukemic cell proliferation was observed in both xenograft models (SEM, RS4;11). DEC therapy responses were detectable early using BLI, preceding changes in leukemic blast frequency in PB.

In contrast and to our surprise, in vivo RS4;11 results were not in line with in vitro results as leukemic proliferation was also diminished in DEC-treated RS4;11 xenograft mice. The difference between in vitro and in vivo results regarding DEC susceptibility of RS4;11 cells might be explained by different application schemes. While DEC was applied only once in vitro, mice were treated daily for 4 days. Repeated DEC applications might be required due to the rapid and irreversible decomposition of this compound and the longer cell doubling time of RS4;11 compared to SEM cells. To test this hypothesis, we calculated the doubling times of cells in SEM-ffluc and RS4;11-ffluc xenografts based on bioluminescence data from days 7, 10, and 14 in saline-treated mice. Of interest, RS4;11 cells exhibited a lower cell doubling time (ranging from 19 to 45 h) in mice than in cell culture (ranging from 51 to 64 h). The proliferation of SEM cells in xenografts was in accordance to our in vitro proliferation data. This might explain the differences between our in vitro and in vivo observations.

Unexpectedly, in vivo concomitant treatment of DEC and AraC did not enhance the antiproliferative effect compared to DEC monotherapy. However, these observations were not consistent with our in vitro results. The combinatorial effect of HMA with Doxo was not studied on BCP-ALL xenografts because NSG mice did not tolerate Doxo dose as published by Ma el al [43]. In our Doxo dose finding study, all Doxo-treated mice loss weight, died during therapy, or were killed by euthanasia due to their bad general state (data not shown).

Further, we demonstrated that DEC also inhibited leukemia cell proliferation in PDX mice. The best therapeutic response was observed in PDX mice of patient #152. Responses in DEC-treated xenografts of #122 and #159 were lower. This could be due to the presence of additional unfavorable gene mutations such as *KRAS* and *JAK3* [44].

In a previous study in AML and MDS patients, DEC leveled out *TP53* mutations associated adverse survival to rates similar to intermediate-risk patients [45]. However, not all patients with TP53 mutations were invariably sensitive to DEC and resistant clones emerged [45].

In addition, we studied the impact on ^18^F-FDG uptake using small animal PET/CT in a BCP ALL xenograft model.^18^F-FDG PET/CT is increasingly used for diagnosis, staging, and evaluation of therapeutic response of several types of cancer including lymphoma [46–48]. So far, it has not been implemented in the assessment of leukemia. Case reports have demonstrated the potential of ^18^F-FDG-PET/CT in the follow-up of leukemic BM infiltration [49–51].

Here, we have shown that ^18^F-FDG-PET/CT is technically feasible and uptake correlates with leukemia expansion. All observations were consistent with BLI data. Our results indicate that ^18^F-FDG-PET/CT might be an applicable method for non-invasive detection of ALL cell metabolism in vivo.

## Conclusions

In conclusion, our series of experiments indicate that HMA are active in MLL-positive BCP-ALL. Simultaneous application of DEC with AraC seems to evoke best outcomes in vitro and not in vivo. We further demonstrated that B-ALL cells can be detected in vivo by ^18^F-FDG-PET/CT raising the possibility of non-invasive response detection. DEC treatment did not eradicate ALL but delayed disease progression in xenograft models. Further studies are required to evaluate therapy responses after repeated DEC applications in BCP-ALL xenografts.

## Additional files

Additional file 1: Patient characteristics. (DOCX 17 kb)

Additional file 2: Methylation-specific quantitative PCR. (MSqPCR) (DOCX 15 kb)

Additional file 3: DEC reduces methylation of LINE-1 and CDH13. (DOCX 49 kb)

Additional file 4: List of SEM-ffluc xenograft mice used for imaging studies. (DOCX 16 kb)

Additional file 5: List of RS4;11-ffluc xenograft mice. (DOCX 14 kb)

Additional file 6: Effects of HMA on proliferation. (DOCX 114 kb)

Additional file 7: Effects of HMA and cytostatic drug combinations on proliferation. (DOCX 231 kb)

Additional file 8: ^18^F-FDG uptake parameter. (DOCX 43 kb)

## Abbreviations

^18^F-FDG: Fluorodeoxyglucose
ALL: Acute lymphoblastic leukemia
AraC: Cytarabine
AZA: Azacitidine
BCP-ALL: B cell precursor acute lymphoblastic leukemia
DEC: Decitabine
DOXO: Doxorubicin
ffluc: Firefly luciferase
GFP: Green fluorescent protein
HMA: Hypomethylating agents
MLL: Mixed lineage leukemia
NSG: NOD scid gamma
PDX: Patient-derived xenograft
PET/CT: Positron emission tomography/computed tomography
