# Supplementary material for: Decitabine demonstrates antileukemic activity in B cell precursor acute lymphoblastic leukemia with MLL rearrangements
Source: J Hematol Oncol. 2018 May 4;11:62. doi: 10.1186/s13045-018-0607-3 (PMC5936021; doi:10.1186/s13045-018-0607-3)
Supplement: Supplementary file 1 — Patient characteristics. (DOCX 17 kb) [file 13045_2018_607_MOESM1_ESM.docx]

**Additional file 1: Patients characteristics**

| **Patient ID** | **Diagnosis** | | **Subtype** | **Gender** | **Age** | **Cytogenetic** | **Cancer**  **Hotspot Mutation*** | **CD19**  **(after ficoll)** | **PDX ID**  **+ Therapy** | | **Days of expansion**  **(in vivo)** |  |
| --- | --- | --- | --- | --- | --- | --- | --- | --- | --- | --- | --- | --- |
| #122 | Initial | common ALL | | male | 47 | t(4;11)(q21;q23), +X,-9, +21 | APC  KRAS  FLT3  TP53 | 83.1 % | PDX-29 | Saline | 29 | |
|  |  |  |  |  |  |  |  |  | - | - |  |  |
|  |  |  |  |  |  |  |  |  | PDX-30 | DEC |  |  |
|  |  |  |  |  |  |  |  |  | PDX-31 | DEC |  |  |
| #152 | Initial | common ALL | | female | 52 | t(4;11)(q21;q23) | HRAS  TP53 | 93.2 % | PDX-33 | Saline | 53 | |
|  |  |  |  |  |  |  |  |  | PDX-35 | Saline |  |  |
|  |  |  |  |  |  |  |  |  | PDX-34 | DEC |  |  |
|  |  |  |  |  |  |  |  |  | PDX-36 | DEC |  |  |
| #159 | Initial | common ALL | | female | 74 | t(4;11)(q21;q23) | NRAS  PIK3CA  TP53  JAK3 | 94.9 % | PDX-26 | Saline | 31 | |
|  |  |  |  |  |  |  |  |  | - | - |  |  |
|  |  |  |  |  |  |  |  |  | PDX-25 | DEC |  |  |
|  |  |  |  |  |  |  |  |  | PDX-27 | DEC |  |  |
