# Supplementary material for: Decitabine demonstrates antileukemic activity in B cell precursor acute lymphoblastic leukemia with MLL rearrangements
Source: J Hematol Oncol. 2018 May 4;11:62. doi: 10.1186/s13045-018-0607-3 (PMC5936021; doi:10.1186/s13045-018-0607-3)
Supplement: Supplementary file 2 — Methylation-specific quantitative PCR. (MSqPCR) (DOCX 15 kb) [file 13045_2018_607_MOESM2_ESM.docx]

**Additional file 2: *Methylation specific quantitative PCR (MSqPCR)***

Genomic DNA from HMA-treated BCP-ALL cells was extracted using the NucleoSpin® Tissue Kit (Machery-Nagel, Dueren, Germany). Bisulfite conversion of DNA was performed with peqGOLD Bisulfite Conversion Kit (VWR Peqlab, Erlangen, Germany) according to the manufacturer’s protocols.

Briefly, MSqPCR was performed in a final volume of 25 µl containing 20-50 ng bisulfite treated DNA, specific primer pairs (Table 1) for unmethylated or methylated CDH13 (0.25 µM) or LINE-1 (0.75 µM) and Quantitect SYBR Green PCR Master Mix (Qiagen, Hilden, Germany). Amplification was performed in biological and technical triplicates using the ViiA7 instrument (Life Technologies, Darmstadt, Germany) as follows: initial denaturation (15 min, 95°C) followed by 45 cycles of denaturation (15 s, 94°C), annealing (30 s, 55°C) and elongation (30 s, 72°C).

The ratio of methylated to unmethylated targets was calculated as 2^∆Ct^, where ∆Ct is the difference between the cycle threshold (Ct) values for the methylated and unmethylated amplicon. Demethylation was calculated using the following formula: *unmethylated [%] = 100 % / (2^ΔCT^ + 1)*.

**Primer sequences**

| **Primer** | **Sequence** |
| --- | --- |
| LINE-1_forw_unmethylated | 5‘ TGTGTGTGAGTTGAAGTAGGGT 3’ |
| LINE-1_rev_unmethylated | 5’ ACCCAATTTTCCAAATACAACCATCA 3’ |
| LINE-1_forw_methylated | 5’ CGCGAGTCGAAGTAGGGC 3’ |
| LINE-1_rev_methylated | 5’ ACCCGATTTTCCAAATACGACCG 3’ |
| CDH13_forw_unmethylated | 5’ TTGTGGGGTTTGTTTTTTGT 3’ |
| CDH13_rev_unmethylated | 5’ AACATTTTCATTCATACACACA 3’ |
| CDH13_forw_methylated | 5’ TCGCGGGGTTCGTTTTTCGC 3’ |
| CDH13_rev_methylated | 5’ GACGTTTTCATTCATACACGCG 3’ |
