# Supplementary material for: Decitabine demonstrates antileukemic activity in B cell precursor acute lymphoblastic leukemia with MLL rearrangements
Source: J Hematol Oncol. 2018 May 4;11:62. doi: 10.1186/s13045-018-0607-3 (PMC5936021; doi:10.1186/s13045-018-0607-3)

**Additional file 3: DEC reduces methylation of LINE-1 and CDH13**

SEM and RS4;11 were exposed to 1,000 nM AZA (a) or 1,000 nM DEC (b) for up to 48 h. Methylation of CDH13 and LINE-1 was evaluated by MSqPCR. In DEC-treated SEM cells methylation was significantly reduced compared to control cells (DMSO=100%) with strongest effects on LINE-1. Experiments were carried out in biological and technical triplicates. Significant treatment effects vs. DMSO control are labeled with * (p<0.05).


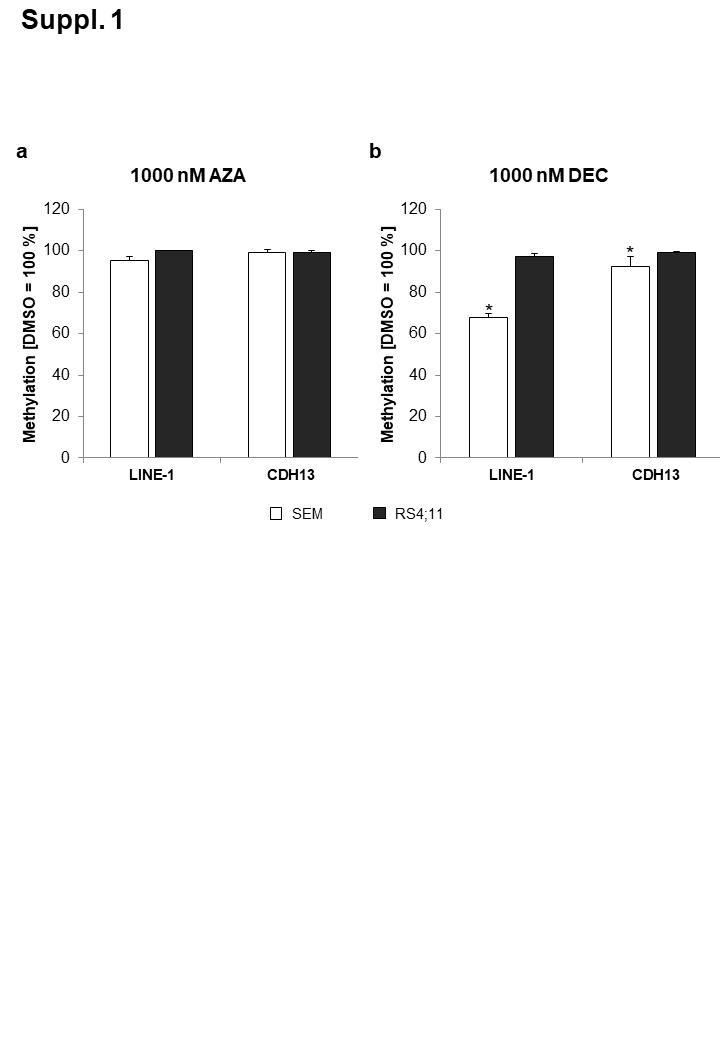

Supplement: Supplementary file 3 — DEC reduces methylation of LINE-1 and CDH13. (DOCX 49 kb) [file 13045_2018_607_MOESM3_ESM.docx]
