# Supplementary material for: Decitabine demonstrates antileukemic activity in B cell precursor acute lymphoblastic leukemia with MLL rearrangements
Source: J Hematol Oncol. 2018 May 4;11:62. doi: 10.1186/s13045-018-0607-3 (PMC5936021; doi:10.1186/s13045-018-0607-3)
Supplement: Supplementary file 4 — List of SEM-ffluc xenograft mice used for imaging studies. (DOCX 16 kb) [file 13045_2018_607_MOESM4_ESM.docx]

| **Treatment** | **Mouse ID** | **BLI** | **PET/CT (day 21)** | **PET/CT (day 28)** | **Final study endpoint**  **[days]** |
| --- | --- | --- | --- | --- | --- |
| **Saline** | NSG-41 | x | x | x | d30 |
|  | NSG-43 | x | x | x | d30 |
|  | NSG-47 | x | - | - | d30 |
|  | NSG-48 | x | x | x | d31 |
|  | NSG-49 | x | - | - | d31 |
|  | NSG-50 | x | - | - | d30 |
|  | NSG-56 | x | - | - | d30 |
|  | NSG-57 | x | x | x | d31 |
|  | NSG-58 | x | - | - | d31 |
| **Decitabine** | NSG-31 | x | - | - | †d14: anesthesia |
|  | NSG-32 | x | - | x | d30 |
|  | NSG-35 | x | - | x | d30 |
|  | NSG-38 | x | - | - | d30 |
|  | NSG-44 | x | - | - | †d16: unexpected |
|  | NSG-52 | x | x | x | d31 |
|  | NSG-53 | x | - | - | d30 |
|  | NSG-59 | x | x | x | d31 |
|  | NSG-60 | x | - | - | d31 |
| **Decitabine + AraC** | NSG-33 | x | - | x | †d28: anesthesia |
|  | NSG-34 | x | - | x | †d28: anesthesia |
|  | NSG-36 | x | - | - | d30 |
|  | NSG-40 | x | - | - | d30 |
|  | NSG-45 | x | - | - | †d16: unexpected |
|  | NSG-46 | x | x | x | d30 |
|  | NSG-54 | x | x | x | d31 |
|  | NSG-55 | x | - | - | d30 |
|  | NSG-61 | x | x | x | d31 |

**Additional file 4: List of SEM-ffluc xenograft mice used for imaging studies**

BLI: bioluminescence; † sudden death
