# Supplementary material for: Decitabine demonstrates antileukemic activity in B cell precursor acute lymphoblastic leukemia with MLL rearrangements
Source: J Hematol Oncol. 2018 May 4;11:62. doi: 10.1186/s13045-018-0607-3 (PMC5936021; doi:10.1186/s13045-018-0607-3)
Supplement: Supplementary file 5 — List of RS4;11-ffluc xenograft mice. (DOCX 14 kb) [file 13045_2018_607_MOESM5_ESM.docx]

**Additional file 5: List of RS4;11-ffluc xenograft mice**

| **Treatment** | **Mouse ID** | **BLI** | **Final study endpoint [days]** |
| --- | --- | --- | --- |
| **Saline** | NSG-106 | x | †d22: anesthesia |
|  | NSG-107 | x | 30 |
|  | NSG-108 | x | 31 |
|  | NSG-114 | x | 30 |
|  | NSG-116 | x | 30 |
|  | NSG-117 | x | 31 |
|  | NSG-124 | x | †d17: anesthesia |
| **Decitabine** | NSG-104 | x | †d22: anesthesia |
|  | NSG-105 | x | †d22: anesthesia |
|  | NSG-112 | x | d30 |
|  | NSG-113 | x | 30 |
|  | NSG-118 | x | 30 |
|  | NSG-119 | x | 31 |
|  | NSG-125 | x | 30 |
|  | NSG-126 | x | 31 |

BLI: bioluminescence; † sudden death
