# Supplementary material for: Decitabine demonstrates antileukemic activity in B cell precursor acute lymphoblastic leukemia with MLL rearrangements
Source: J Hematol Oncol. 2018 May 4;11:62. doi: 10.1186/s13045-018-0607-3 (PMC5936021; doi:10.1186/s13045-018-0607-3)
Supplement: Supplementary file 6 — Effects of HMA on proliferation. (DOCX 114 kb) [file 13045_2018_607_MOESM6_ESM.docx]

**Additional file 6: Effects of HMA on proliferation**

SEM and RS4;11 cells were exposed to HMA for up to 72 h. Cell numbers were determined by counting viable cells using trypan blue dye exclusion. Displayed are mean values ± standard deviations of three independent experiments.
