# Supplementary material for: Decitabine demonstrates antileukemic activity in B cell precursor acute lymphoblastic leukemia with MLL rearrangements
Source: J Hematol Oncol. 2018 May 4;11:62. doi: 10.1186/s13045-018-0607-3 (PMC5936021; doi:10.1186/s13045-018-0607-3)
Supplement: Supplementary file 7 — Effects of HMA and cytostatic drug combinations on proliferation. (DOCX 231 kb) [file 13045_2018_607_MOESM7_ESM.docx]

**Additional file 7: Effects of HMA and cytostatic drug combinations on proliferation**

Cells were treated with HMA in the absence or presence of AraC **(a)** or Doxo **(b)**. Cytostatic drugs (CTX) were added simultaneously, 24 h before or 24 h after treatment with HMA. CTX concentrations were based on Iow doses and used as follows: 100 nM AZA (SEM), 500 nM AZA (RS4;11), 100 nM DEC (SEM), 500 nM DEC (RS4;11); 10 nM AraC (SEM); 2500 nM AraC (RS4;11), 12.5 nM Doxo (SEM, RS4;11). The proliferation was determined by cell count 72 h after drug application. Results were expressed as a percentage of DMSO-treated control cells and displayed as mean ± SD of three independent experiments.
