# Supplementary material for: Decitabine demonstrates antileukemic activity in B cell precursor acute lymphoblastic leukemia with MLL rearrangements
Source: J Hematol Oncol. 2018 May 4;11:62. doi: 10.1186/s13045-018-0607-3 (PMC5936021; doi:10.1186/s13045-018-0607-3)

**Additional file 8:** **^18^F-FDG uptake parameter**

PET/CT was performed on days 21 and 28 in SEM-ffluc xenografts.

^18^F-FDG uptake in spleen is calculated for all mice and expressed as metabolic max [% ID/g] and metabolic tumor volume [mm^3^]. Results are summarized as mean ± SD.


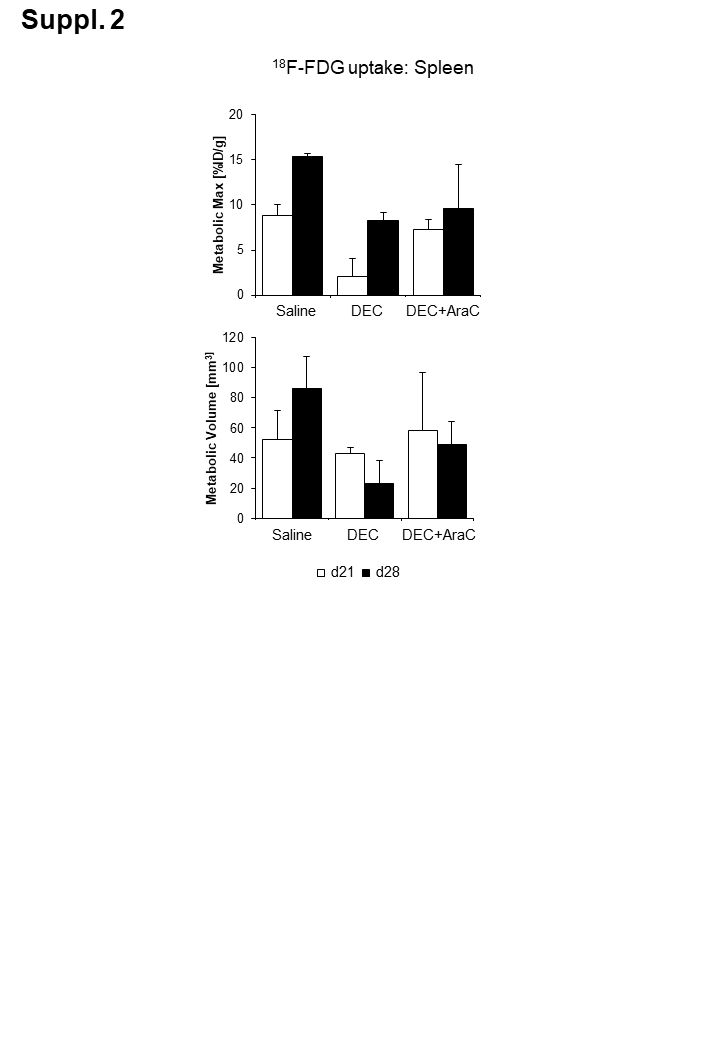

Supplement: Supplementary file 8 — 18F-FDG uptake parameter. (DOCX 43 kb) [file 13045_2018_607_MOESM8_ESM.docx]
